# Rhamnolipids and surfactin inhibit the growth or formation of oral bacterial biofilm

**DOI:** 10.1186/s12866-020-02034-9

**Published:** 2020-11-23

**Authors:** Ryota Yamasaki, Aki Kawano, Yoshie Yoshioka, Wataru Ariyoshi

**Affiliations:** Division of Infections and Molecular Biology, Department of Health Promotion, Kyushu Dental University, Kitakyushu, Fukuoka 803-8580 Japan

**Keywords:** Biofilm inhibition, Oral bacteria, Rhamnolipids, Surfactin

## Abstract

**Background:**

Bacteria survive in various environments by forming biofilms. Bacterial biofilms often cause significant problems to medical instruments and industrial processes. Techniques to inhibit biofilm formation are essential and have wide applications. In this study, we evaluated the ability of two types of biosurfactants (rhamnolipids and surfactin) to inhibit growth and biofilm formation ability of oral pathogenic bacteria such as *Aggregatibacter actinomycetemcomitans*, *Streptococcus mutans*, and *Streptococcus sanguinis*.

**Results:**

Rhamnolipids inhibited the growth and biofilm formation ability of all examined oral bacteria. Surfactin showed effective inhibition against *S. sanguinis* ATCC10556, but lower effects toward *A. actinomycetemcomitans* Y4 and *S. mutans* UA159. To corroborate these results, biofilms were observed by scanning electron microscopy (SEM) and confocal microscopy. The observations were largely in concordance with the biofilm assay results. We also attempted to determine the step in the biofilm formation process that was inhibited by biosurfactants. The results clearly demonstrated that rhamnolipids inhibit biofilm formation after the initiation process, however, they do not affect attachment or maturation.

**Conclusions:**

Rhamnolipids inhibit oral bacterial growth and biofilm formation by *A. actinomycetemcomitans* Y4, and may serve as novel oral drug against localized invasive periodontitis.

**Supplementary Information:**

The online version contains supplementary material available at 10.1186/s12866-020-02034-9.

## Background

Biofilms are three-dimensional structures consisting of various microbial populations attached to a surface by extracellular polymeric substances (EPS) produced by these microorganisms [1]. Bacteria are physically shielded from external stresses by this extracellular matrix, [2]. Although biofilms can be beneficial in industrial processes such as wastewater treatment [3], fermentation [4], and microbial fuel cells [5, 6], they can also cause harmful effects, including biocorrosion by sulfate-reducing bacteria [7], infection due to biofilm formation on medical devices, such as catheters, pacemakers, and artificial joints [8], and oral diseases such as tooth decay and periodontal disease [9]. *Streptococcus mutans*, a representative bacteria of tooth decay [10], forms plaques (biofilm) on tooth surfaces and produces lactate from sugars such as sucrose, which demineralizes teeth and causes dental caries. *Streptococcus sanguinis* is another plaque-forming strain that has been reported to cause infective endocarditis by forming biofilms on the endocardium and heart valves [11]. *Aggregatibacter actinomycetemcomitans* has been strongly associated with localized aggressive periodontitis via its ability to form biofilms in the subgingival cavity [12]. Apart from causing oral diseases, these pathogenic bacteria also cause systemic diseases, such as arteriosclerosis [13] and diabetes [14]. Therefore, removal of biofilms formed by periodontopathogenic bacteria prevents a variety of diseases. However, oral care by brushing has been insufficient in healthy adults, much less infants and the elderly. Denture wearing and treatment by an orthodontist may also be insufficient. Although physical methods are fundamental for removing oral biofilms, there is a wide variation in its efficiency due to individual differences. Anti-bacterial agents and disinfectants are effective against planktonic bacteria (bacteria suspended in liquid), however, they are not effective against biofilm-forming bacteria due to the difficulty of chemicals penetrating the biofilm. Hence, there is concern about the risk of the emergence of resistant bacteria, such as the formation of persister cells [15–18]. Oral pathogens that form persisters may cause recurrence of oral diseases via regrowth. Therefore, inhibiting the growth of oral pathogens, as well as effectively preventing biofilm formation contribute to reducing these risks. Thus, there is a requirement for the development of a simpler and more effective oral care capable of inhibiting oral bacterial growth and biofilm formation.

EPS is a basic component of biofilms comprising polysaccharides, enzymes, DNA, lipids, and various other factors [19, 20], which determine the physicochemical properties of biofilms formed by bacteria [21, 22]. Strains such as *Pseudomonas aeruginosa* produce biosurfactants, including rhamnolipids [23]. Rhamnolipids have a rhamnose sugar moiety and are linked to alkanoic acid fatty acid tails such as myrmicacin [24]. Rhamnolipids exhibit cytotoxicity as hemolysins [25], and participate in bacterial communication as a quorum-sensing substance [26]. They have also been found to be dispersed in the biofilms of various bacteria, such as *Bordetella bronchiseptica* [27] and *Desulfovibrio vulgaris* [28]. Surfactin is another type of biosurfactant secreted by *Bacillus subtilis* [29]. Its structure includes a hydrophilic cyclic peptide consisting of seven amino acids and a hydrophobic hydrocarbon chain [30]. Surfactin is a signaling molecule that initiates biofilm formation [29]. In contrast, there is a report that surfactin inhibits biofilms of *Salmonella enterica* [31]. Hence, both of these types of biosurfactants are associated with bacterial biofilm formation. Therefore, it can be hypothesized that they have significant inhibitory effects against biofilms formed by oral pathogenic bacteria.

However, inhibiting oral pathogenic bacterial growth and biofilm formation is important not only for suppressing oral diseases, but also for ameliorating systemic diseases. Therefore, in this study, we investigated the inhibitory effects of biosurfactants (rhamnolipids and surfactin) against bacterial growth, and biofilm formation by *A. actinomycetemcomitans* Y4, *S. mutans* UA159, and *S. sanguinis* ATCC10556. Further, we investigated which process required for biofilm formation (attachment, initiation, or maturation) is inhibited by biosurfactants.

## Results

### Rhamnolipids and surfactin exhibit variable inhibitory effects on bacterial cell growth

**Fig. 1:**
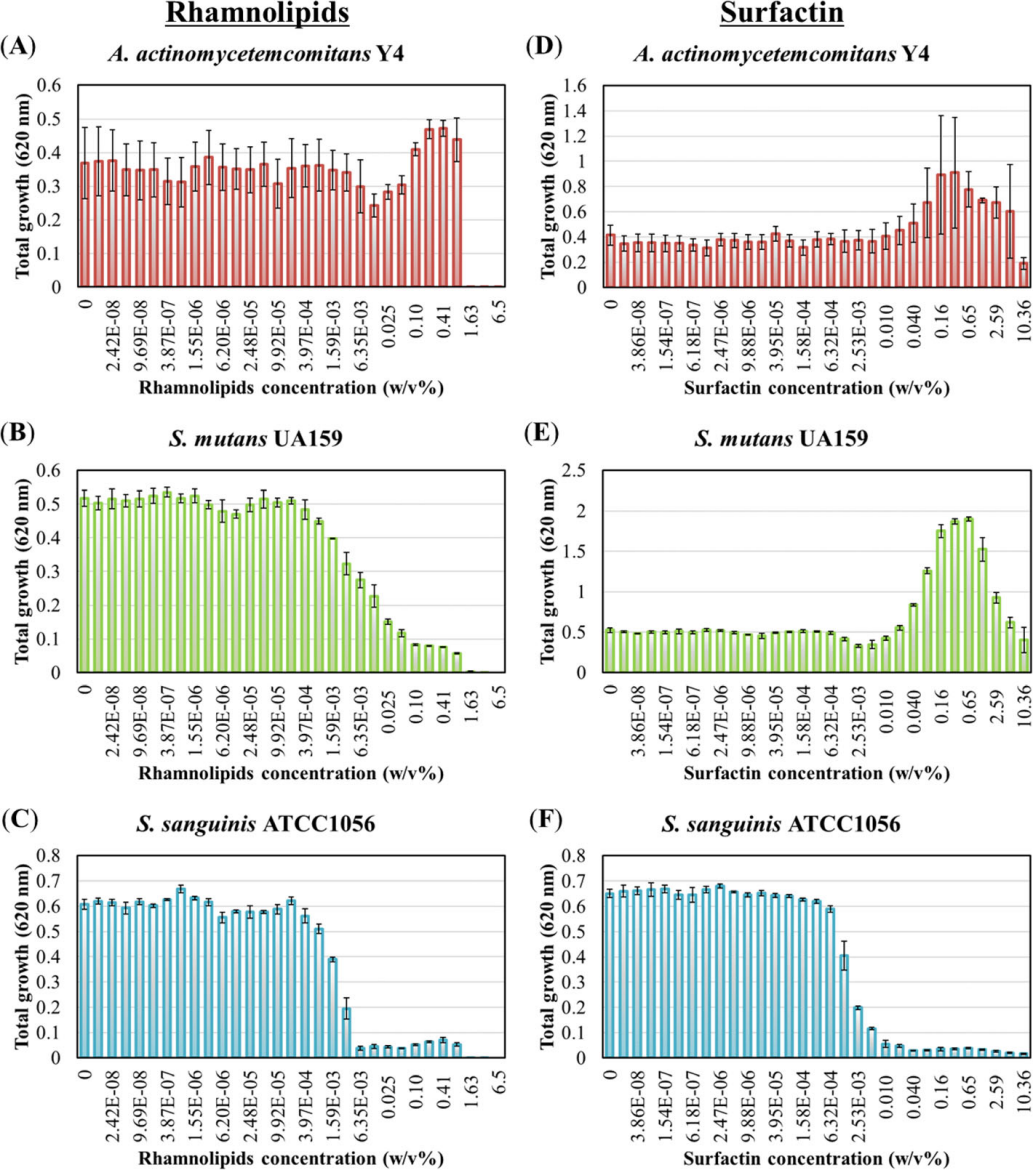
Growth inhibition of oral pathogenic bacteria by biosurfactants. Total growth of (**a**, **d**) *A. actinomycetemcomitans* Y4, (**b**, **e**) *S. mutans* UA159, and (**c**, **f**) *S. sanguinis* ATCC10556 by rhamnolipids (**a-c**) or surfactin (**d-f**). Rhamnolipid was used at a final concentration ranging from 6.5 w/v% to 1.21 × 10^− 8^ w/v% (a 2-fold serial dilution was applied) in BHI broth. Surfactin was used at a final concentration ranging from 10.36 w/v% to 1.93 × 10^− 8^ w/v% (a 2-fold serial dilution was applied) in BHI broth. Error bars indicate standard deviations of at least three experiments

First, the ability of rhamnolipids and surfactin to inhibit the growth of bacteria was investigated. The results showed that rhamnolipids significantly inhibited the growth of *S. mutans* UA159 and *S. sanguinis* ATCC10556, however, *A. actinomycetemcomitans* Y4 was not affected (Fig. 1a-c). Rhamnolipids completely inhibited the growth of *A. actinomycetemcomitans* Y4 at a concentration of 3.25 w/v% (Fig. 1a). However, rhamnolipids inhibited both *S. mutans* UA159 and *S. sanguinis* ATCC10556 growth at concentrations > 1.59 × 10^− 3^ w/v%, whereas these two bacterial species were nearly completely inhibited at 3.25 w/v% and 6.35 × 10^− 3^ w/v% rhamnolipid, respectively (Fig. 1b and c). Alternatively, surfactin exhibited the highest inhibitory effect on *S. sanguinis* ATCC10556, whereas no effect was observed on *A. actinomycetemcomitans* Y4 and *S. mutans* UA159 (Fig. 1d-f). Moreover, high concentrations of surfactin promoted *A. actinomycetemcomitans* Y4 and *S. mutans* UA159 growth, rather than inhibited it (Fig. 1d and e). Nevertheless, at a concentration > 1.26 × 10^− 3^ w/v%, surfactin inhibited *S. sanguinis* ATCC10556 growth, with near complete inhibition observed at 0.01 w/v% (Fig. 1f).

#### Biosurfactants exhibit variable inhibitory effects on biofilm formation

**Fig. 2:**
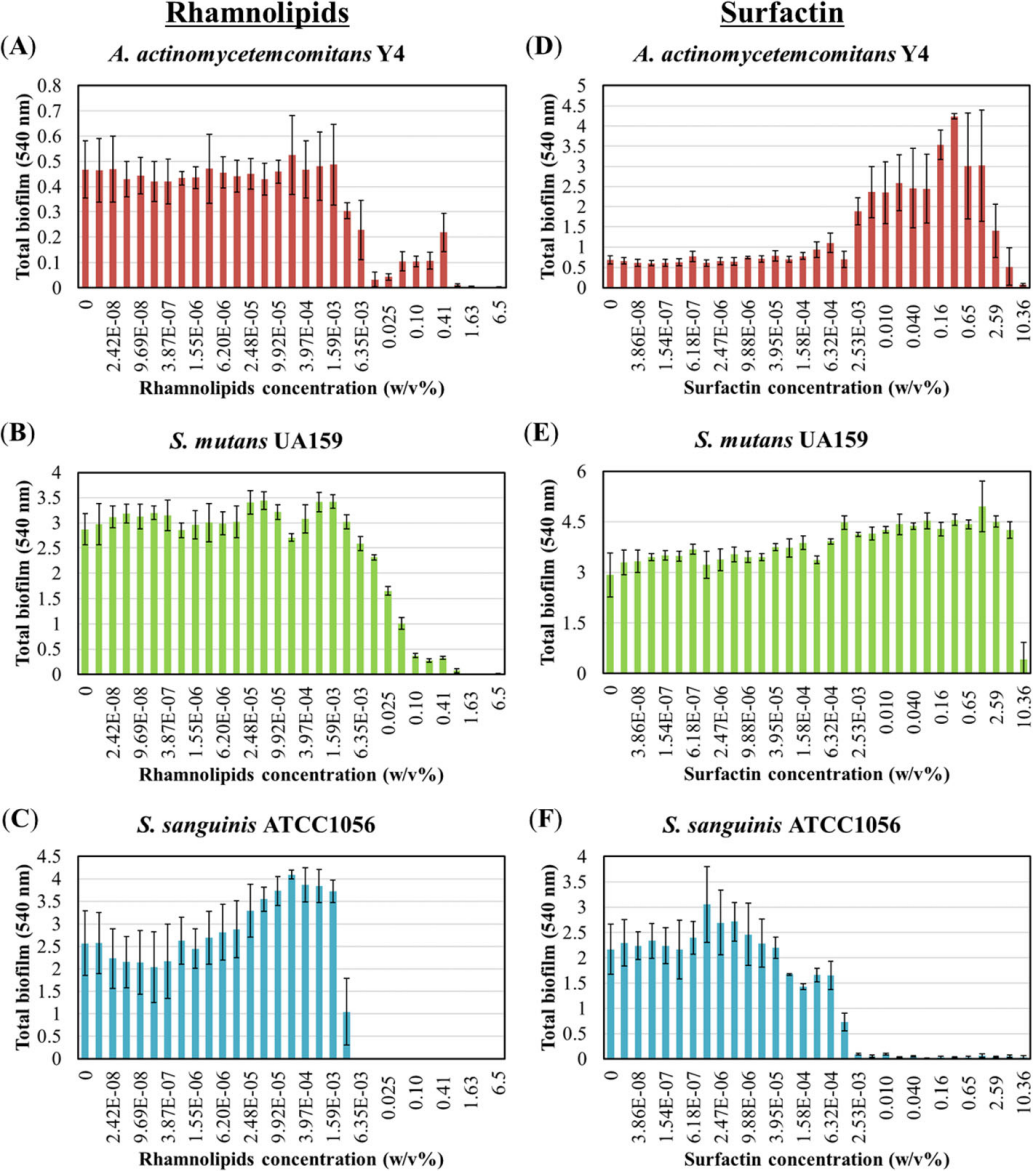
Inhibition of biofilm formation by biosurfactants. Total biofilm formation of (**a**, **d**) *A. actinomycetemcomitans* Y4, (**b**, **e**) *S. mutans* UA159, and (**c**, **f**) *S. sanguinis* ATCC10556 by rhamnolipids (**a-c**) or surfactin (**d-f**). Rhamnolipids was used at a final concentration ranging from 6.5 w/v% to 1.21 × 10^− 8^ w/v% (a 2-fold serial dilution was applied) in BHI broth. Surfactin was used at a final concentration ranging from 10.36 w/v% to 1.93 × 10^− 8^ w/v% (a 2-fold serial dilution was applied) in BHI broth. Error bars indicate standard deviations of at least three experiments

The inhibitory capacity of biosurfactants on biofilm formation is depicted in Fig. 2. At 3.17 × 10^− 3^ w/v%, rhamnolipids exhibited inhibitory effects against *A. actinomycetemcomitans* Y4 biofilm formation, while at 0.013 w/v%, biofilm formation was inhibited by 93% (Fig. 2a). Interestingly, although *A. actinomycetemcomitans* Y4 growth was not inhibited at rhamnolipid concentrations < 0.81 w/v%, biofilm formation was inhibited from 0.81 w/v% to 3.17 × 10^− 3^ w/v% (Figs. 1a and 2a). Hence, low concentrations of rhamnolipids were capable of only inhibiting biofilm formation without affecting bacterial growth. In case of *S. mutans* UA159, rhamnolipids at a concentration of 6.35 × 10^− 3^ w/v% inhibited biofilm formation, however, near complete inhibition was observed at 0.1 w/v% (Fig. 2b). Meanwhile, in *S. sanguinis* ATCC10556, rhamnolipids completely inhibited biofilm formation at a concentration of 6.35 × 10^− 3^ w/v%. Alternatively, rhamnolipids promoted biofilm formation (2-fold) at 1.98 × 10^− 4^ w/v% (Fig. 2c). Furthermore, approximately 90% of *A. actinomycetemcomitans* Y4 biofilm formation was inhibited at surfactin concentration of 10.36 w/v%. However, 2.53 × 10^− 3^ w/v% to 2.59 w/v% promoted *A. actinomycetemcomitans* Y4 biofilm formation by up to 6-fold (Fig. 2d). Meanwhile, treatment of *S. mutans* UA159 with surfactin promoted biofilm formation with no inhibitory effect observed at any concentration (Fig. 2e). Finally, concentrations > 2.53 × 10^− 3^ w/v% of surfactin caused near complete inhibition of *S. sanguinis* ATCC10556 biofilm formation (Fig. 2f).

#### Scanning electron microscopy and confocal microscopy analyses confirm the effect of biosurfactants on biofilm formation

**Fig. 3:**
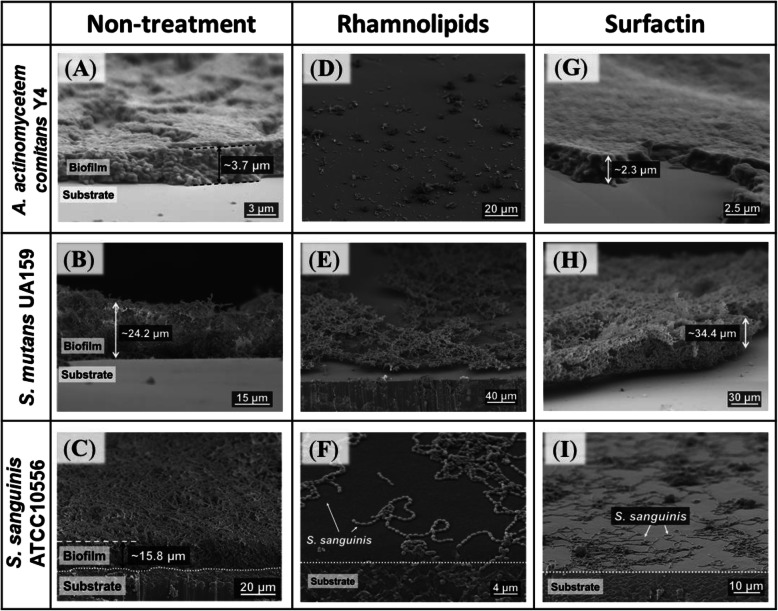
Biofilm observation. Representative images of *A. actinomycetemcomitans* Y4 (**a**, **d, g**), *S. mutans* UA159 (**b, e, h**), and *S. sanguinis* ATCC10556 (**c, f, i**) biofilms as visualized by SEM. The SEM images for the following samples is shown: untreated control (0 w/v%), rhamnolipid treatment (0.65 w/v%), and surfactin treatment (1.04 w/v%)

Next, the thickness of the oral pathogenic bacterial biofilms with or without biosurfactant treatment (0.65 w/v% rhamnolipids and 1.04 w/v% surfactin) was estimated by scanning electron microscopy (SEM) (Fig. 3) and confocal microscopy (Supplemental Fig. 1). As shown in the SEM images, untreated *A. actinomycetemcomitans* Y4, *S. mutans* UA159, and *S. sanguinis* ATCC10556 formed thick biofilms (3–25 μm; Fig. 3a-c). Meanwhile, at a concentration of 0.65 w/v% rhamnolipid, biofilm formation by *A. actinomycetemcomitans* Y4 and *S. sanguinis* ATCC10556 was almost completely inhibited (Fig. 3d and f) and that of *S. mutans* UA159 was moderately inhibited (Fig. 3e). Surfactin at 1.04 w/v% induced high levels of inhibition against *S. sanguinis* ATCC10556 biofilm formation (Fig. 3i); however, it did not show an inhibitory effect against *A. actinomycetemcomitans* Y4 and *S. mutans* UA159 biofilm formation (Fig. 3g and h).

Observations from all fluorescence confocal microscopy images (Supplemental Fig. 1) were in concordance with the SEM results. Similar tendencies were observed when comparing Figs. 2 and 3.

#### To determine the step of biofilm formation inhibited by rhamnolipids

**Fig. 4:**
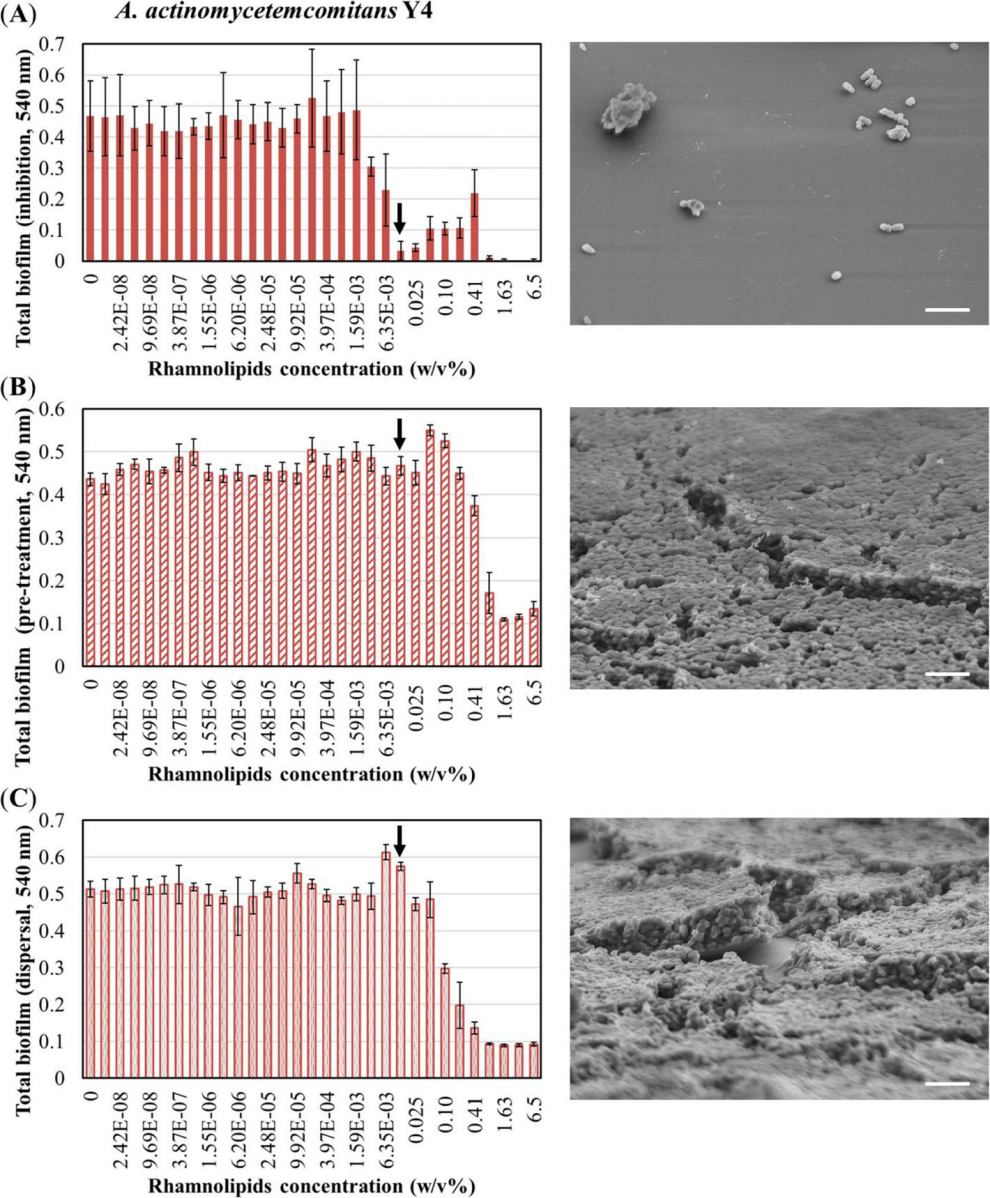
Effect of rhamnolipids on each step of biofilm formation. **a** Graphical representation of the total biofilm formed by *A. actinomycetemcomitans* Y4 treated with rhamnolipids (same as Fig. 2**a**). The image on the right shows SEM image of *A. actinomycetemcomitans* Y4 biofilms after incubation with 0.013 w/v% rhamnolipid. **b** Graphical representation of the total *A. actinomycetemcomitans* Y4 biofilm formed on pre-treated rhamnolipid microtiter plate. This assay demonstrates inhibition of the attachment step of biofilm formation. The image on the right shows SEM image of *A. actinomycetemcomitans* Y4 biofilm upon pre-treatment with 0.013 w/v% rhamnolipid. **c** Graphical representation of the total *A. actinomycetemcomitans* Y4 biofilm formed after rhamnolipid dispersal. This assay demonstrates the effect of rhamnolipids against matured biofilm. The image on the right shows SEM images of *A. actinomycetemcomitans* Y4 biofilms after dispersal treatment with 0.013 w/v% rhamnolipid. Black arrows indicate the rhamnolipid concentration that exhibited highest inhibitory effect in (**a**) (0.013 w/v%). Error bars indicate standard deviations of at least three experiments. Scale bars indicate 3 μm

To investigate the step of biofilm formation the rhamnolipids exert their inhibitory effect on (attachment, initiation, or maturation process), pre-treatment for the attachment process and dispersal for the maturation process were performed. As rhamnolipids exhibited the highest inhibitory effect against *A. actinomycetemcomitans* Y4 biofilm formation (Fig. 2a), rhamnolipid-treatment conditions were examined. As observed earlier, rhamnolipids affected the step of biofilm formation between initiation (after attachment) and maturation as it was co-cultured with rhamnolipids (Fig. 4a left = Fig. 2a). Rhamnolipids exhibited inhibitory effects against *A. actinomycetemcomitans* Y4 biofilm formation at 3.17 × 10^− 3^ w/v%. Significant inhibitory effects were observed beginning at 0.013 w/v% of rhamnolipids (Fig. 4a left, black arrow). Although *A. actinomycetemcomitans* Y4 biofilm was observed by SEM after rhamnolipid treatment at this concentration, biofilms were nearly completely inhibited (Fig. 4a right). Meanwhile, Fig. 4b presents the results of the rhamnolipid pre-treatment test, wherein the attachment process was investigated. Specifically, plates were pre-treated with rhamnolipids, after which the attachment of bacteria was detected. At concentrations higher than 0.81 w/v%, pre-treatment with rhamnolipids inhibited *A. actinomycetemcomitans* Y4 biofilm formation, however, < 0.41 w/v% did not elicit an effect (Fig. 4b left). The SEM image (Fig. 4b right) indicated that pretreatment at 0.013 w/v% of rhamnolipids resulted in no inhibition on biofilm formation. Next, the effect of rhamnolipids on the maturation of *A. actinomycetemcomitans* Y4 biofilms was investigated. In this assay, rhamnolipids were added to the biofilm after 24 h of culturing; hence the biofilm was already established with attachment and initiation having already occurred. This assay, therefore, examined the effect on the final stage of the biofilm life cycle, i.e., dispersion, during which cells leave the biofilm to become planktonic, thereby making themselves more susceptible to antimicrobials. Results show that although rhamnolipids induced biofilm dispersal at concentrations > 0.1 w/v%, this effect was not observed at concentrations < 0.05 w/v% (Fig. 4c left). This effect on dispersal was also observed by SEM after treatment with 0.013 w/v% rhamnolipid, whereby the biofilms were not removed (Fig. 4c right).

## Discussions

In this study, we investigated the effect of biosurfactants (rhamnolipids and surfactin) on the growth of pathogenic bacteria and biofilm formation. The results showed that rhamnolipids showed high anti-bacterial and anti-biofilm effects against oral bacteria, particularly *Streptococcus* spp. (Figs. 1a-c and 2a-c). Similarly, numerous previous studies have reported anti-bacterial and anti-biofilm activities of rhamnolipids. For example, *Listeria monocytogenes* growth is reportedly inhibited at a concentration of 0.25 w/v% [32]. Meanwhile, growth inhibition has been reported for *Bacillus cereus* and *Staphylococcus aureus* at 6.4 × 10^− 3^ w/v% and 25.6 × 10^− 3^ w/v% of rhamnolipids [33]. Additionally, for various bacterial pathogens including *Enterobacter aerogenes* and *Klebsiella pneumoniae*, growth inhibition was noted at concentrations ranging from 0.5 × 10^− 3^ w/v% to 0.25 w/v% [34–37]. Herein, the growth of *A. actinomycetemcomitans* Y4, *S. mutans* UA159, and *S. sanguinis* ATCC10556 was completely inhibited at 3.25 w/v%, 3.25 w/v%, and 6.35 × 10^− 3^ w/v%, respectively (Fig. 1a-c). Hence, rhamnolipids exhibited high inhibitory effects against the growth of cariogenic bacteria.

Furthermore, an anti-biofilm effect of rhamnolipids has been previously reported for *Pseudomonas aeruginosa* at 5 × 10^− 3^ w/v% [38]. Similarly, a 60% inhibitory effect was observed for *Staphylococcus capitis* and *Bacillus licheniformis* at a rhamnolipid concentration of 4 × 10^− 3^ w/v% [39]. Meanwhile, our current results demonstrated that rhamnolipids inhibited *A. actinomycetemcomitans* Y4 biofilm formation by 93% at 0.013 w/v%, (Fig. 2a), and inhibited *S. mutans* UA159 by 87% at 0.1 w/v% (Fig. 2b). Additionally, *S. sanguinis* ATCC10556 biofilm formation was completely inhibited at concentrations > 6.35 × 10^− 3^ w/v% (Fig. 2c). Hence, we confirmed that all examined oral pathogenic bacteria were inhibited by rhamnolipids at concentrations similar to those previously reported. Moreover, considering that rhamnolipids also inhibited the growth of *S. mutans* UA159 and *S. sanguinis* ATCC10556, the inhibitory effect on biofilms was likely due to bacterial growth inhibition (Fig. 1a-c). Alternatively, rhamnolipids inhibited *A. actinomycetemcomitans* Y4 biofilm formation, however, they did not inhibit bacterial growth at concentrations ranging from 3.17 × 10^− 3^ w/v% to 0.81 w/v% (Figs. 1a and 2a). Therefore, rhamnolipids likely directly inhibited *A. actinomycetemcomitans* Y4 biofilm formation. However, the potential of rhamnolipids to inhibit *A. actinomycetemcomitans* Y4 biofilm formation decreased at 0.41 w/v%, which was confirmed in six different experimental replicates. Although the precise cause of this result is unknown, it is apparent that only specific concentrations of rhamnolipids elicit inhibitory effects on *A. actinomycetemcomitans* Y4 biofilm formation.

A number of studies have also reported anti-bacterial and anti-biofilm activity of surfactin toward various bacteria. For example, growth inhibition of *Staphylococcus epidermidis* was reported at a surfactin concentration of 0.625 w/v% [40]. Additionally, 0.5 × 10^− 3^ w/v% of surfactin inhibited the growth of sulfate-reducing bacteria (*Desulfovibrio alaskensis*) [41]. Herein, we confirmed that surfactin inhibits growth of specific oral pathogens, particularly *S. sanguinis* ATCC10556 (Fig. 1f). At concentrations > 1.26 × 10^− 3^ w/v%, surfactin inhibited *S. sanguinis* ATCC10556 growth; moreover, nearly complete inhibition was observed at 0.01 w/v%, indicating that surfactin is capable of inhibiting bacterial species to a level similar to that previously reported [40, 41]. However, surfactin also caused growth promotion of *A. actinomycetemcomitans* Y4 and *S. mutans* UA159 (Fig. 1d and e). Production of surfactin by *Bacillus subtilis* serves to enhance bacterial growth and for biological control activity via direct antagonism of pathogens [29]. Moreover, considering that the structure of surfactin contains a peptide chain [30], it may function as a nutrient and growth promotor.

The anti-biofilm activity of surfactin has also been demonstrated against various bacteria. For example, 6.6 × 10^− 3^ w/v% of surfactin eliminated *Legionella pneumophila* biofilms [42]. Surfactin also reportedly inhibits the formation of *L. monocytogenes*, *Enterobacter sakazakii*, and *Salmonella* Enteritidis biofilms on stainless steel and polypropylene surfaces [43]. Herein, we found that surfactin only inhibited biofilm formation of specific oral pathogens. Nearly 100% of *S. sanguinis* ATCC10556 biofilm formation was inhibited by 2.53 × 10^− 3^ w/v% surfactin treatment (Fig. 2f). However, no inhibitory effect was observed in the other two oral bacteria (*A. actinomycetemcomitans* Y4 and *S. mutans* UA159), rather an enhancing effect was noted (Fig. 2d and e). We, therefore, postulate that surfactin enhanced biofilm formation by these two bacterial species via enhancing the growth of the individual cells, as described earlier.

To identify which biofilm process was inhibited by biosurfactants, the attachment and maturation steps were assessed. A number of previous studies have indicated that pre-treatment with rhamnolipids effectively inhibits biofilm formation. For example, pre-treatment of polystyrene surfaces with 1 w/v% rhamnolipids caused a 58 and 68% inhibitory effect on the formation of *L. monocytogenes* and *S aureus* biofilms, respectively [44]. Additionally, the adhesion of *B. licheniformis* was inhibited by 85% following pre-treatment with 9 × 10^− 3^ w/v% rhamnolipids [39]. Herein, we observed that pre-treatment with > 0.81 w/v% rhamnolipids effectively inhibited more than 63% of *A. actinomycetemcomitans* Y4 biofilm formation (Fig. 4b left), thereby confirming its inhibitory effect on the attachment process. Although rhamnolipids inhibited more than 90% of *A. actinomycetemcomitans* Y4 biofilm formation at 0.013 w/v% (Fig. 4a left, black arrow), pre-treatment with 0.013 w/v% resulted in no inhibitory effect (Fig. 4b left, black arrow and right SEM image). Therefore, it may be inferred that the inhibitory effect exhibited by rhamnolipids does not interfere with bacterial cell adhesion. Next, the dispersal effect of rhamnolipids on mature biofilms was investigated. Previous reports employing a rhamnolipid-deficient bacterial strain demonstrated the role of rhamnolipid on biofilm dispersal. The authors observed that biofilms formed by sulfate-reducing bacteria can be dispersed by rhamnolipids present in the supernatant of *Pseudomonas aeruginosa* PA14 [28, 45]. The current study demonstrated 43–83% biofilm dispersion following treatment with > 0.1 w/v% rhamnolipids. However, 0.013 w/v% rhamnolipids did not impact maturation of the biofilm (Fig. 4c left, black arrow and SEM image on the right panel). Therefore, the primary effect exhibited by rhamnolipids does not appear to be associated with the maturation process. Taken together, these results indicate that the inhibitory effect of biosurfactants, specifically, rhamnolipids, toward the formation of biofilms by oral pathogenic bacteria, targets a stage after attachment, from initiation to maturation. Moreover, considering that *A. actinomycetemcomitans* Y4 grew well following treatment with 0.013 w/v% rhamnolipids, in spite of the inhibition of biofilm formation (Fig. 2a), the anti-biofilm effect was not dependent on bactericidal activity. However, the quorum-sensing system is strongly associated with biofilm maturation and dispersion [46, 47]. Specifically, autoinducer-2, known as the only quorum-sensing molecule in *A. actinomycetemcomitans*, is closely related to biofilm formation [48–50]. Hence, biosurfactants may interfere with the quorum-sensing system by suppressing biofilm formation. However, the specific details regarding this mechanism require further investigation. Specifically, the use of bacterial strains deficient in various quorum-sensing molecules, including quormones, may serve to further decipher the mechanism by which biosurfactants affect quorum-sensing and biofilm formation.

Currently, there is need for improved oral health practices to reduce the growth of pathogenic oral bacteria and associated biofilms. To this end, various kinds of compound (chlorhexidine [51], povidone iodine [52], hydrogen peroxide [53], acidified sodium chlorite [54], and cetylpyridinium chloride [55]) are used as mouthwash agents. However, these compounds have various associated risks, including diabetes [56] and oral cancer [57]. Moreover, chlorhexidine has reportedly caused anaphylaxis [58], whereas oral ulcerations have been observed following the use of hydrogen peroxide [59]. Furthermore, acidified sodium chlorite has been reported to cause enamel erosion similar to orange juice [60], and cetylpyridinium chloride induces cell death via the apoptotic pathway [61]. Hence, components of common mouthwash agents have adverse side effects associated with them. Alternatively, biosurfactants (rhamnolipids and surfactin) have low toxicity and are eco-friendly [62, 63], and may, therefore, provide a superior option for improved oral care. Rhamnolipids, specifically, may serve as an optimal choice as a preventive medicine for periodontal disease as it demonstrated strong anti-bacterial and anti-biofilm activity against *A. actinomycetemcomitans* Y4.

There are some limitations to this study. First, the mechanisms of inhibition of growth and biofilm formation by biosurfactants were not elucidated. We suggested that the inhibitory activity of rhamnolipids against *A. actinomycetemcomitans* Y4 does not involve the attachment and maturation process of biofilm formation. However, the exact mechanism and step at which the process is inhibited remains unclear. To overcome this limitation, mutants for biofilm-associated genes may be employed to investigate the mechanism of biofilm inhibition. Additionally, the relationship between quorum-sensing molecules and biosurfactants may be deduced as quorum-sensing system is closely related to biofilm formation. Second, the oral cavity is a complex environment, comprising of a wide variety of bacterial species either as planktonic cells or incorporated into biofilms. In the present study, we demonstrated the effect of biosurfactants toward a single strain of cariogenic and periodontopathic bacteria. It is also important to conduct experiments in an environment closer to the oral cavity to verify the combined effect of the gamut of bacterial flora present in this region. In-depth analysis regarding the associated risks of using biosurfactants in the oral cavity, including clinical trials, are required to verify their safety profile. Clinical studies on healthy subjects or patients with localized invasive periodontitis caused by *A. actinomycetemcomitans* Y4 are warranted to validate biosurfactants as a medicine for periodontal disease.

## Conclusion

In this study, we demonstrated the inhibitory effect of biosurfactants (rhamnolipids and surfactin) against oral bacterial pathogens. Specifically, we found that rhamnolipids exhibited both anti-bacterial and anti-biofilm activity against *A. actinomycetemcomitans* Y4. Both biosurfactants showed a high inhibitory effect against *S. sanguinis* ATCC10556 growth. Rhamnolipids showed high inhibitory effect against growth of *S. mutans* UA159 and *S. sanguinis* ATCC10556 and high biofilm inhibitory effects against *A. actinomycetemcomitans* Y4. In addition, we propose that rhamnolipids primarily interfere with *A. actinomycetemcomitans* Y4 biofilm formation after the attachment or maturation step. However, elucidation of the detailed mechanism is required. Owing to their low toxicity and eco-friendly properties, rhamnolipids and surfactin can serve as potential medications for the treatment and prevention of oral diseases. From this study, we suggest the possibility of rhamnolipids to be used as a preventive agent for localized invasive periodontitis caused by *A. actinomycetemcomitans* Y4.

## Methods

### Bacteria cultivation

The strains used in this study are listed in Table 1. *A. actinomycetemcomitans* Y4, *S. mutans* UA159, and *S. sanguinis* ATCC10556 were cultured in brain heart infusion broth (BHI: Becton Dickinson, Heidelberg, Germany) containing 1% (w/v) yeast extract at 37 °C in a 5% CO_2_ atmosphere.

**Table 1:** Bacterial strains used in this study

Strains	Source
*A. actinomycetemcomitans* Y4	ATCC43718 [64]
*S. mutans* UA159	ATCC700610 [65]
*S. sanguinis* ATCC10556	ATCC10556

### Biofilm assay with biosurfactant

Rhamnolipids (AGAE Technologies, LLC, OR, USA) and sodium surfactin (Kaneka, Osaka, Japan) were dissolved into BHI medium at concentrations of 6.5 and 10.36 w/v%, respectively. A series of 2-fold serial dilution solutions were prepared into 96-well microtiter plate (i.e. in case of rhamnolipids, first rows are 6.5 w/v%, second rows are 3.25 w/v%, third rows are 1.63 w/v%, and last rows are 1.21 × 10^− 8^ w/v%). Sucrose (0.1 w/v%) for *S. mutans* UA159, and 1 w/v% sucrose for *S. sanguinis* ATCC10556 were added to all wells to form biofilms (Supplemental Fig. 2). A saturated culture of each oral strain was inoculated into all wells to a turbidity of 0.05 at 600 nm, except for the blank. These plates were incubated at 37 °C in a 5% CO_2_ atmosphere for 24 h to form biofilms. After 24 h incubation, total growth was measured at 620 nm. Next, the supernatant was discarded, and the plate was washed with dH_2_O 3 times to remove planktonic cells. Crystal violet (0.1%) was added to stain the biofilm for 20 min and subsequently removed by washing the plate with dH_2_O 3 times; next, 95% ethanol was added. After a 5 min incubation, the plate was measured at an absorbance of 540 nm using a microplate reader (Multiskan FC, Thermo Fisher Scientific, Waltham, MA, USA).

### Microscopic observation of oral biofilms

SEM (S-4300, HITACHI, Tokyo, Japan) and confocal microscope (BZ-X800, Keyence, Osaka, Japan) were employed to observe each oral pathogenic bacterial biofilm. Oral pathogenic bacteria with or without biosurfactants were incubated into ibidi μ-Plate 96 square well plate (NIPPON Genetics Co., Ltd., Tokyo, Japan) at 37 °C in a 5% CO_2_ atmosphere for 24 h to form biofilms. The supernatant was discarded, and the plate was washed with dH_2_O 3 times to remove planktonic cells. Glutaraldehyde was used as a fixative for biofilms to prepare SEM observation samples. Biofilms were dehydrated by ethanol gradient (50, 70, 90, 99, and 100% anhydrous with molecular sieves). After dehydration, ethanol was replaced by t-butanol and freeze dried at − 20 °C. Frosted t-butanol was sublimated by lyophilization. The biofilm sample was then coated by Pt spattering (Magnetron sputter MSP-1S, Vacuum Device Inc., Ibaraki, Japan). Accelerating voltages were performed at 5.0 kV and magnifications were adjusted X350–6000. To prepare the confocal microscopy samples, biofilms were stained with SYTO9 for 15 min in the dark. Biofilms were washed with PBS and air-dried in the dark at room temperature. The excitation wavelength of SYTO9 was 450–490 nm, and the emission was 500–550 nm. The fluorescence images were analyzed using the BZ-H4A software (Keyence, Osaka, Japan).

### Biofilm pre-treatment and dispersal assay

For pre-treatment with rhamnolipids, 200 μL of rhamnolipids dissolved in 1X PBS (concentration ranging from 6.5 w/v% to 1.21 × 10^− 8^ w/v%) were added into a 96-well microtiter plate and incubated at 37 °C in a 5% CO_2_ atmosphere for 1 h. As a control, 200 μL of 1X PBS was used (i.e. 0 w/v%). Rhamnolipids solutions were discarded and 200 μL of *A. actinomycetemcomitans* Y4 culture (OD_600_ ~ 0.05) was added into all pre-treated wells. After 24 h incubation at 37 °C in a 5% CO_2_ atmosphere, biofilm assays were performed using the same method as mentioned above.

To investigate biofilm dispersal, 200 μL of *A. actinomycetemcomitans* Y4 culture (OD_600_ ~ 0.05) was added into a 96-well microtiter plate and incubated at 37 °C, 5% CO_2_ for 24 h. The supernatant was discarded and washed with dH_2_O thrice to remove planktonic cells. Rhamnolipids dissolved in 1X PBS (200 μL each and concentration ranging from 6.5 w/v% to 1.21 × 10^− 8^ w/v%, and 0 w/v% as a control) were added into 96-well microtiter plates, which contained *A. actinomycetemcomitans* Y4 biofilms, and incubated at 37 °C in a 5% CO_2_ atmosphere for 1 h. After 1 h incubation, biofilm assays were performed according to the same method as mentioned above.

## Supplementary Information

**Additional file 1:** **Supplemental Fig. 1.** Representative images of *A. actinomycetemcomitans* Y4 (**A**, **D, G**), *S. mutans* UA159 (**B, E, H**), and *S. sanguinis* ATCC10556 (**C, F, I**) biofilms as visualized by confocal microscopy. Non-treatment (0 w/v%) images (**A-C**), rhamnolipids treatment (0.65 w/v%) images (**D-F**), and surfactin treated (1.04 w/v%) images (**G-H**) are shown. These bacterial samples were stained by Syto9. Scale bars indicates 10 μm. **Supplemental Fig. 2.** Biofilm formation of (**A**) *S. mutans* UA159 and (**B**) *S. sanguinis* ATCC10556 with sucrose concentrations (0, 0.01, 0.1, and 1 w/v%). Error bars indicate standard deviations of at least three experiments.

## Data Availability

All data generated or analyzed during this study are included in this published article and its supplementary information files.

## Abbreviations

SEM: Scanning electron microscopy
EPS: Extracellular polymeric substances
DNA: Deoxyribonucleic acid
BHI: Brain heart infusion
dH_2_O: Distilled water
PBS: Phosphate buffered saline
OD: Optical density
