# Supplementary material for: Rhamnolipids and surfactin inhibit the growth or formation of oral bacterial biofilm
Source: BMC Microbiol. 2020 Nov 23;20:358. doi: 10.1186/s12866-020-02034-9 (PMC7684882; doi:10.1186/s12866-020-02034-9)
Supplement: Supplementary file 1 — Additional file 1: Supplemental Fig. 1. Representative images of A. actinomycetemcomitans Y4 (A, D, G), S. mutans UA159 (B, E, H), and S. sanguinis ATCC10556 (C, F, I) biofilms as visualized by confocal microscopy. Non-treatment (0 w/v%) images (A-C), rhamnolipids treatment (0.65 w/v%) images (D-F), and surfactin treated (1.04 w/v%) images (G-H) are shown. These bacterial samples were stained by Syto9. Scale bars indicates 10 μm. Supplemental Fig. 2. Biofilm formation of (A) S. mutans UA159 and (B) S. sanguinis ATCC10556 with sucrose concentrations (0, 0.01, 0.1, and 1 w/v%). Error bars indicate standard deviations of at least three experiments. [file 12866_2020_2034_MOESM1_ESM.docx]

**SUPPORTING INFORMATION**

**Rhamnolipids and surfactin inhibit oral bacterial biofilms formation**

**Ryota Yamasaki**^*^, **Aki Kawano, Yoshie Yoshioka, Wataru Ariyoshi**

Division of Infections and Molecular Biology, Department of Health Promotion, Kyushu Dental University, Kitakyushu, Fukuoka 803-8580 Japan

*For correspondence:

E-mail: r18yamasaki@fa.kyu-dent.ac.jp

Tel. (+81) 93-285-3051

**Running title:** Oral bacterial biofilms are inhibited by biosurfactant.

**Keywords:** biofilm inhibition, oral bacteria, supernatant, rhamnolipids, surfactin


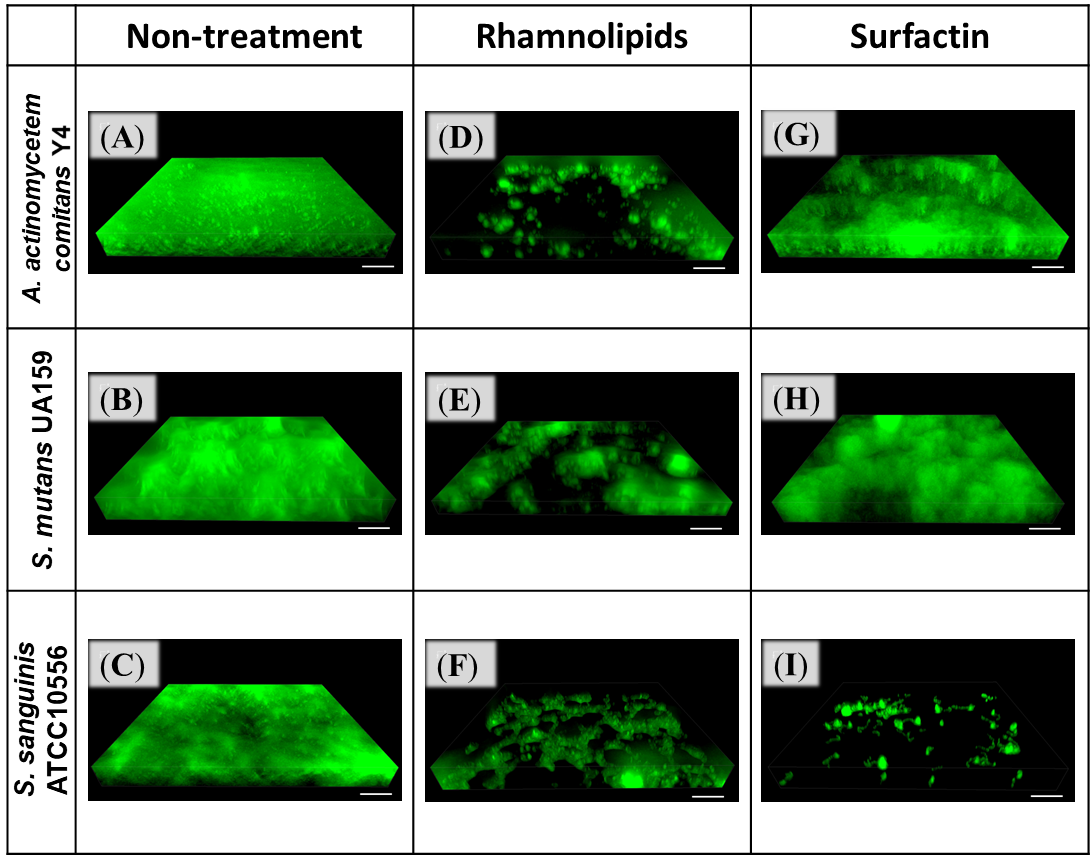


**Supplemental Fig. 1.** Representative images of *A. actinomycetemcomitans* Y4 (**A**, **D, G**), *S. mutans* UA159 (**B, E, H**), and *S. sanguinis* ATCC10556 (**C, F, I**) biofilms as visualized by confocal microscopy. Non-treatment (0 w/v%) images (**A-C**), rhamnolipids treatment (0.65 w/v%) images (**D-F**), and surfactin treated (1.04 w/v%) images (**G-H**) are shown. These bacterial samples were stained by Syto9. Scale bars indicates 10 µm.


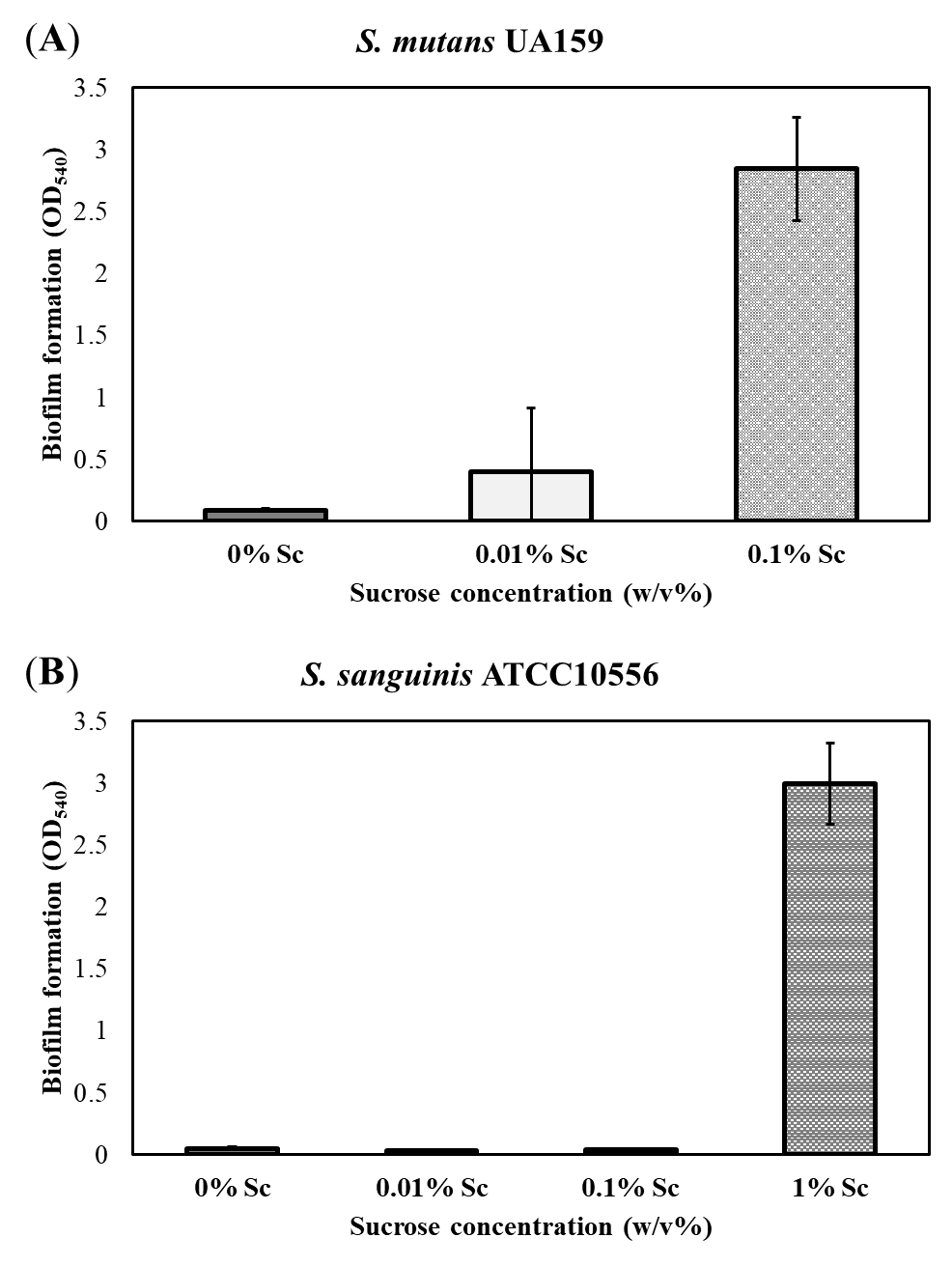


**Supplemental Fig. 2.** Biofilm formation of (**A**) *S. mutans* UA159 and (**B**) *S. sanguinis* ATCC10556 with sucrose concentrations (0, 0.01, 0.1, and 1 w/v%). Error bars indicate standard deviations of at least three experiments.
